# Late-presenting congenital diaphragmatic hernias in children: A single-center case series over 16 years

**DOI:** 10.1097/MD.0000000000045533

**Published:** 2025-11-21

**Authors:** Ayoung Kang, Soo-Hong Kim, Hae-Young Kim

**Affiliations:** a Division of Pediatric Surgery, Department of Surgery, Pusan National University Children’s Hospital, Pusan National University Yangsan Hospital, Yangsan, Republic of Korea; b Department of Surgery, School of Medicine, Pusan National University, Busan, Republic of Korea; c Research institute for Convergence of Biomedical Science and Technology, Pusan National University Yangsan Hospital, Yangsan, Republic of Korea.

**Keywords:** case series, children, congenital diaphragmatic hernia, late-presentation, surgical outcomes

## Abstract

Congenital diaphragmatic hernia (CDH) typically manifests as respiratory distress during the neonatal period, and is associated with high mortality rates. However, late-presenting CDH that occurs after the neonatal period is rare and often leads to misdiagnosis and delayed treatment, potentially resulting in severe complications. This study aimed to review our experience with late-presenting CDH cases to better understand its characteristics and facilitate earlier diagnosis and treatment. We retrospectively analyzed the medical records of 62 patients aged <18 years who underwent CDH surgery between 2008 and 2024 at Pusan National University Children’s Hospital, Korea. Patients in the neonatal period and those with diaphragmatic eventration, hiatal hernias, or morgagni hernias were excluded. We evaluated demographics, symptoms, associated anomalies, surgical findings, and outcomes. Over a 16-year period, seven patients underwent surgery for late-presenting CDH at our center. Among them, 5 were male (71%) and 2 were female (29%). The median patient age at the time of surgery was 9 months. Four patients (57%) presented with respiratory symptoms, including cough and dyspnea, whereas the remaining 3 (43%) exhibited gastrointestinal symptoms, such as nausea and vomiting. Diagnosis was achieved in 6 patients (86%) through computed tomography and in 1 patient (14%) through ultrasonography. Three patients with gastrointestinal symptoms due to bowel obstruction or gastric volvulus and 1 patient with respiratory distress caused by lung compression from herniated abdominal organs underwent emergency surgery. Three patients with upper respiratory tract infections underwent surgery after the infections resolved. Primary repair without patch placement was successfully performed in all patients, with a 100% survival rate. Late-presenting CDH can manifest with a wide range of gastrointestinal or respiratory symptoms. When children present with unexplained gastrointestinal or respiratory issues, late-presenting CDH should be considered as a differential diagnosis. Although the number of cases is limited, timely diagnosis and appropriate surgical treatment may lead to favorable outcomes.

## 1. Introduction

Congenital diaphragmatic hernia (CDH) is a rare anomaly characterized by a defect in the diaphragm that results in the migration of abdominal organs into the thoracic cavity. CDH can present in various locations and with different symptoms; however, the most common form is Bochdalek hernia, which involves a defect in the posterolateral aspect of the diaphragm. Most patients with CDH exhibit respiratory distress due to lung compression caused by herniated abdominal organs, leading to pulmonary hypoplasia and development of pulmonary hypertension within a few hours of birth. Despite advances in medical technology, neonatal CDH still has a high mortality rate. Therefore, CDH remains a critical condition, particularly during the neonatal period.^[1–3]^

Some cases of CDH are diagnosed after the neonatal period, which is known as late-presenting CDH. The reported incidence of late-presenting CDH varies widely in the literature, ranging from 5% to 45% of all CDH cases. In general, late-presenting CDH is associated with a relatively favorable prognosis compared with neonatal-onset CDH.^[4]^ However, its diagnosis is often challenging owing to diverse gastrointestinal and respiratory symptoms. Moreover, prompt diagnosis and immediate treatment are crucial because delayed recognition and management can result in severe complications, including death.^[2,5,6]^

This study aimed to report and analyze the various symptoms of late-presenting CDH to facilitate its early and accurate diagnosis. We present a retrospective analysis of our center’s experience with late-presenting CDH and a literature review.

## 2. Materials and methods

We retrospectively analyzed the medical records of patients aged under 18 years who underwent CDH surgery between 2008 and 2024 at Pusan National University Children’s Hospital, Korea. Patients within neonatal period at diagnosis and those with diaphragmatic eventration, hiatal hernia, Morgagni hernia, or traumatic hernia were excluded. Seven patients were enrolled in the study. We evaluated the birth history, age at diagnosis, sex, hernia site, presenting symptoms and signs, associated anomalies, diagnostic methods, surgical findings, and postoperative outcomes.

All surgeries were performed under general anesthesia via an open transabdominal approach through a subcostal incision on the affected side. After reducing the herniated abdominal organs into the abdominal cavity, the diaphragmatic defect was closed primarily using Ethibond Excel® (Polybutylate-Coated Polyester, Johnson & Johnson MedTech [New Brunswick]), a nonabsorbable suture material. All diaphragmatic defects were amenable to primary suture repair. No synthetic or biologic patch materials were used in any case. MIS was not performed in any case due to a combination of factors, including the emergent nature of surgery, limited availability of appropriately sized instruments for pediatric patients, and the judgment by the surgical and anesthesia teams that open repair would be more reliable and safe under the given circumstances.

This study was approved by the Institutional Review Board of the Pusan National University Yangsan Hospital (IRB No. 55-2025-033). The requirement of written informed consent to publish this case report was waived by the Institutional Review Board.

## 3. Results

Among the 62 patients who underwent surgery for CDH during the study period, 7 had late-presenting CDH, resulting in an overall incidence of 11%. The main findings of the patients are summarized in Table 1. The median age at diagnosis was 9 months (range: 4 months to 12 years), with 5 males (71%) and 2 females (29%). All patients were born full-term, none had a low birth weight, and prenatal ultrasound examinations revealed no abnormalities. Except for 1 patient (case 5) with lamin muscular dystrophy, no other comorbidities, including cardiac anomalies, were present. All patients had defects in the posterolateral part of the diaphragm, with 5 patients (71%) having left-sided defects and 2 patients (29%) having right-sided defects. The median maximal diameter of the diaphragmatic defect was 4 cm (range: 2.5 –7.0 cm). Both patients with right-sided CDH had hernial sacs composed of very thin membranes. Because the sacs were extremely thin with minimal muscular components, they were clearly distinguishable from diaphragmatic eventrations.

**Table 1:** Summary of the clinical features, operative findings, and outcomes.

Case	Age	Sex	Weight (kg)	Clinical symptoms	Site	Maximal diameter of diaphragmatic defect (cm)	Hernial sac	Herniated organ
Group A: Respiratory symptoms								
1	5 mo	F	7.33	Cough, rhinorrhea, fever for 3 d	Right	4	Present	Liver
2	8 yr	M	42	Dyspnea for 5 d	Left	7	Absent	Small intestine, colon
3	4 mo	M	7.5	Cough for 2 wk	Right	4	Present	Liver
4	12 mo	M	8	Cough, rhinorrhea, fever for 4 d	Left	5	Absent	Stomach, spleen, small intestine, colon
Group B: Gastrointestinal symptoms								
5	9 mo	M	8.6	Vomiting for 3 d	Left	2.5	Absent	Small intestine, colon
6	6 mo	F	8.4	Vomiting for 2 d	Left	3	Absent	Small intestine, colon
7	12 yr	M	63	Vomiting for 2 d	Left	7	Absent	Stomach, spleen, small intestine, colon

### 3.1. Clinical presentation

By symptom presentation, 4 patients (57%) exhibited respiratory symptoms, while 3 patients (43%) presented with gastrointestinal symptoms.

#### 3.1.1. Patients with respiratory symptoms

**Figure 1.**
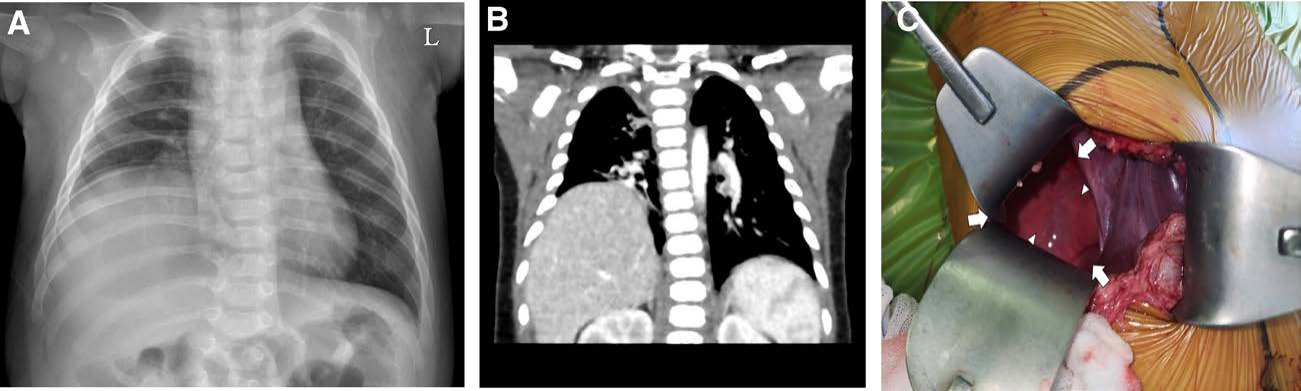
Preoperative imaging and intraoperative findings in case 3. (A) Chest x-ray reveals a focal opacity in the right lower lung zone with an elevated right diaphragmatic margin, initially suggestive of pneumonia. (B) CT scan demonstrates the liver herniating into the thoracic cavity, indicating a diaphragmatic hernia or eventration. (C) Intraoperative findings show a diaphragmatic defect (arrow), with lung tissue visible beyond a thin sac (arrowhead). CT = computed tomography.

**Figure 2.**
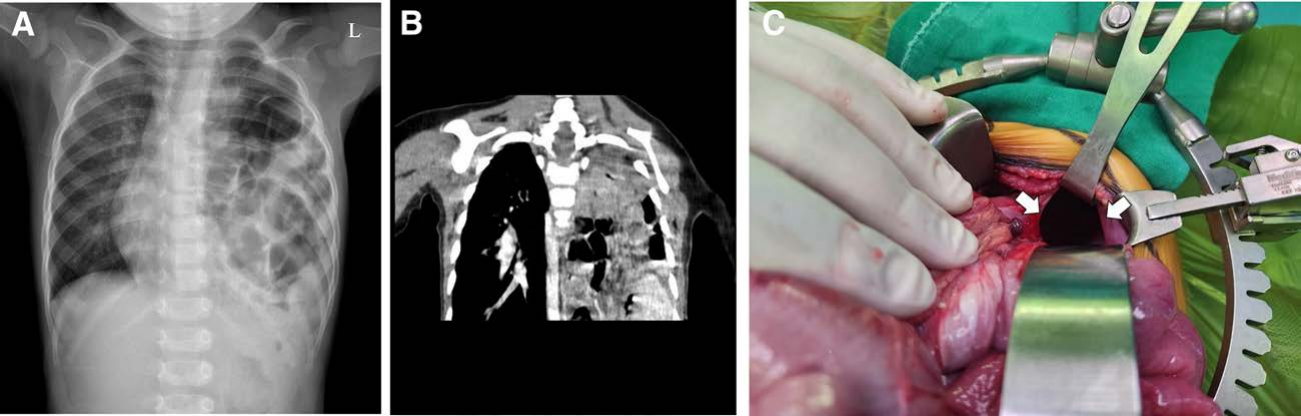
Preoperative imaging and intraoperative findings in case 4. (A) Chest X-ray and (B) CT scan reveal multiple herniated bowel loops in the left thoracic cavity. (C) Intraoperative findings show a diaphragmatic defect (arrow) after the reduction of the herniated organs. CT = computed tomography.

Among those with respiratory symptoms, 3 patients (cases 1, 3, and 4) were initially treated at local clinics for upper respiratory infection symptoms, including cough, rhinorrhea, and fever, for 3, 14, and 4 days, respectively. However, as the symptoms did not improve, they visited our center. All 3 patients had abnormal findings on initial chest x-rays. Late-presenting CDH was diagnosed in 2 patients via CT and in the remaining patients through abdominal and chest ultrasound (Fig. 1). The cause of infection was not identified in cases 1 and 3, whereas respiratory syncytial virus and adenovirus infections were confirmed in case 4. Surgery was performed in all 3 patients after their respiratory infections resolved. Two patients (cases 1 and 3) had right-sided CDH with a hernial sac, including herniation of the right lobe of the liver (Fig. 1). The other patient (case 4) had a left-sided defect with herniation of the stomach, spleen, most of the small intestine, and right colon (Fig. 2). Owing to the presence of intestinal malrotation, Ladd’s procedure and incidental appendectomy were performed simultaneously.

**Figure 3.**
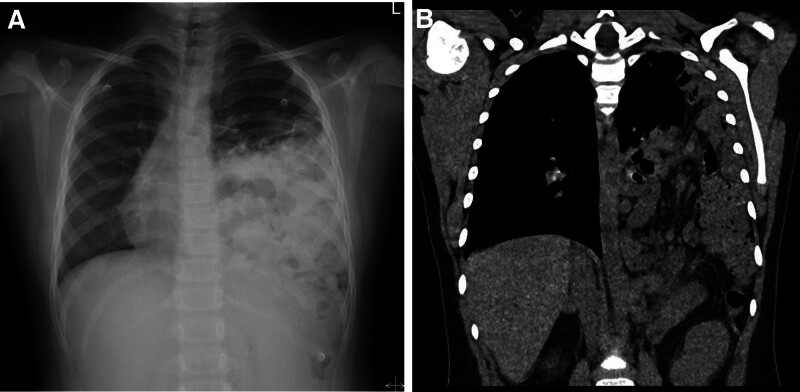
Preoperative imaging in case 2. (A) Chest x-ray shows a hazy shadow in the lower left chest cavity. (B) CT scan reveals a bowel loop herniated into the left thoracic cavity. CT = computed tomography.

Another patient (case 2) with respiratory symptoms visited the emergency room because of progressively worsening dyspnea, which did not improve despite treatment at a local hospital for 5 days. The diagnosis was made using chest radiography and CT (Fig. 3), and emergency surgery was performed because there were no signs of infection; worsening dyspnea was suspected to be caused by lung compression due to herniated abdominal organs. The defect was located on the left side with herniation of the stomach, spleen, small intestine, and part of the right colon.

#### 3.1.2. Patients with gastrointestinal symptoms

**Figure 4.**
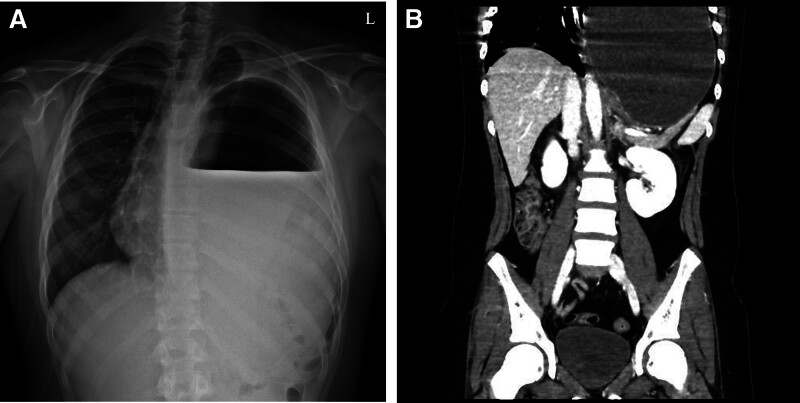
Preoperative imaging in case 7. (A) Chest x-ray shows an opacified left hemithorax with an air-fluid level, mimicking pleural effusion. (B) CT scan reveals a severely distended, herniated stomach within the thoracic cavity. CT = computed tomography.

Patients with gastrointestinal symptoms presented with vomiting lasting 2, 3, or 2 days. Two of them (cases 5 and 6) were suspected of having intestinal obstruction, while the other (case 7) was suspected of having gastric volvulus due to a hernia. Diagnoses were made using chest radiography and CT, and all 3 patients underwent emergency surgery. All the patients had defects in the left posterolateral diaphragm (Fig. 4). Two patients with intestinal obstruction had herniations of parts of the small and large intestines, whereas the patient with gastric volvulus had herniations of the stomach, spleen, small intestine, and part of the right colon.

### 3.2. Diagnostic modalities

Chest radiography was performed on all patients. Six patients underwent computed tomography (CT), while 1 patient underwent abdominal and thoracic ultrasonography for CDH diagnosis. Among patients with respiratory symptoms, 3 were diagnosed via CT and 1 via ultrasonography. In the gastrointestinal group, all diagnoses were confirmed by CT following suggestive chest radiographs. CT was the most commonly used modality, especially in emergent settings or when gastric volvulus or bowel obstruction was suspected.

### 3.3. Operative approach and findings

All patients underwent primary repair as described in the Methods section. None of the patients required patch or implant repair. Right-sided CDH with a hernial sac was noted in 2 patients. Intestinal malrotation was present only in case 4, requiring concurrent Ladd’s procedure and incidental appendectomy.

### 3.4. Postoperative outcomes

All patients received intensive care unit treatment, including intensive monitoring or mechanical ventilation, for 2 to 6 days postoperatively. No immediate postoperative complications were observed. The patients were discharged between the 8th and 13th postoperative days.

The median follow-up period was 11 months (range: 2 months to 8 years). One patient (case 3) developed adhesive ileus requiring hospitalization. The patient’s condition improved after fasting and decompression using a nasogastric tube. None of the other patients experienced any complications. All patients remained well without hernia recurrence or need for reoperation during the follow-up period.

## 4. Discussion

The diaphragm develops between the 8th and 10th weeks of gestation as the pleura, peritoneum, and pleuroperitoneal membranes merge. The diaphragm completely separates the thoracic and abdominal cavities, preventing high abdominal pressure from compressing the thoracic cavity and ensuring proper functioning of the respiratory and circulatory systems. However, abnormalities in diaphragm formation or structural weakness would lead to various diaphragmatic disorders.^[3,7–9]^ Late-presenting CDH is also thought to be caused by these factors; however, the cause of the delayed onset of symptoms is unknown. Acquired herniation of the abdominal organs develops through a congenital diaphragmatic defect that is covered by the spleen or liver.^[1]^

The parents of the patients were often asked when the condition had developed after surgery. If it was congenital, they questioned why it only manifested now rather than at birth. All the patients in this study underwent prenatal ultrasound examinations, and no abnormalities were detected. In addition, some patients had previously undergone chest radiography, which was considered normal. None of the patients had any history of trauma. During surgery, an examination of the diaphragmatic defect revealed a mucosal lining along the margin, which was distinct from the damaged appearance typically observed in traumatic hernias. This finding suggests that traumatic hernia can be ruled out. However, this finding only indicates that the diaphragmatic defects were not recent and does not serve as definitive evidence that the condition was congenital.

Previous studies have suggested that late-presenting CDH is likely a congenital condition owing to the high prevalence of associated anomalies (8.6–80%).^[4,10]^ However, in this study, only 1 patient (14%) had an associated anomaly, which does not strongly support this hypothesis. Nevertheless, in cases such as Case 4, where intestinal malrotation and diaphragmatic hernia were present at a very young age, there would appear to be evidence suggesting a congenital origin. In some cases, associated malformations would go undiagnosed or could be only discovered at autopsy, continued follow-up and further research should be needed.^[11]^

In this study, none of the patients were diagnosed before chest radiography was performed. In some cases, local physicians recommended obtaining chest radiographs because of abnormal auscultation findings. When abnormal breath sounds or atypical respiratory or digestive symptoms are present, chest radiography should be performed promptly, and when the chest radiograph reveals abnormal findings suspicious of late-presenting CDH, performing early CT or ultrasound would be helpful for a rapid diagnosis based on our experience.^[12,13]^ Diagnosis can be made using chest radiography alone; however, misdiagnosis is common, as conditions such as pneumonia, pleural effusion, or pneumothorax are often mistaken for CDH. There has been a reported case in which a chest tube was inserted due to a misdiagnosis of pleural effusion, leading to an iatrogenic stomach injury.^[5]^ Delays in diagnosis and treatment are also associated with mortality. In Korea, there was a case where a physician was sentenced to imprisonment for failing to diagnose late-presenting CDH, which resulted in the patient’s death.^[6,14]^ CT carries a risk of radiation exposure, which carries a risk of associated complications. CT provides high diagnostic accuracy and rapid availability in emergency settings; however, radiation exposure remains a well-recognized concern in children, who are more sensitive to ionizing radiation and have a longer lifetime risk horizon.^[15]^ Nevertheless, because of the wide range of symptoms associated with late-presenting CDH and the potential risk of fatal outcomes in cases with unexplained respiratory or digestive symptoms and abnormal chest radiographic findings, we recommend performing additional tests such as CT at an early stage.

Although ultrasonography is often recommended as a first-line modality for pediatric thoracic abnormalities, it is highly operator-dependent and not always available in urgent settings, especially during nights or weekends. Delaying diagnosis while awaiting ultrasound may be unsafe in children with acute respiratory distress or gastrointestinal obstruction. MRI provides excellent anatomical detail without radiation exposure, but its use is limited in emergencies because of high cost, long acquisition times, and the frequent need for sedation in infants and young children. For these reasons, CT remains the most practical and reliable modality in acute late-presenting CDH, despite concerns about radiation exposure.^[15,16]^

Generally, early surgical intervention is preferred to prevent complications, but the patient’s condition should be considered when determining the timing of surgery.^[4,5,17]^ We performed immediate surgery in 4 patients and delayed surgery while waiting to improve the upper respiratory tract infection in 3 patients. All patients with digestive symptoms such as vomiting underwent emergency surgery. One case of gastric volvulus and 2 cases of intestinal obstruction were considered fatal if delayed. Among the patients with respiratory symptoms, 1 had difficulty breathing due to compression by a herniated organ and underwent immediate surgery.

Since the 3 patients with upper respiratory tract infections did not have dyspnea or digestive symptoms, and their overall condition was stable, we initially believed that the symptoms of cough, sputum, and fever were unrelated to late-presenting CDH. In other words, we assumed that the late-presenting CDH was asymptomatic and incidentally discovered during the treatment for upper respiratory tract infections. Notably, 2 of these patients had right-sided CDH with a hernial sac, which supported the decision to delay surgery. Delaying the operation helped avoid anesthesia-related complications associated with respiratory infections, and the outcomes were favorable.

Minimally invasive surgery (MIS), including laparoscopic and thoracoscopic repair, has been increasingly reported for late-presenting CDH. MIS may offer advantages such as reduced postoperative pain, faster recovery, and superior cosmetic outcomes.^[18,19]^ However, its application in infants and young children is technically challenging because of the small operative field, limited availability of appropriately sized instruments, and the physiological risks of pneumoperitoneum, such as hypercapnia.^[18,20]^ Importantly, safe performance of MIS in this context also requires experienced pediatric anesthesia support, which is not always readily available in emergency settings or outside of large tertiary centers.^[20]^ In our series, open repair was chosen as the most reliable and safe approach under these circumstances. Future multicenter studies are warranted to clarify the role of MIS and to establish evidence-based criteria for patient selection.

This study is a retrospective case series conducted at a single tertiary referral center. Due to the rare nature of late-presenting CDH, the sample size is inevitably small, and clinical presentations may not represent the full spectrum of this condition. Although no recurrence was observed, the follow-up period was heterogeneous and insufficient in some cases to assess long-term prognosis. These factors should be considered when interpreting our findings.

## 5. Conclusion

Patients with late-presenting CDH may present with a broad range of gastrointestinal and respiratory symptoms. When children exhibit unexplained gastrointestinal or respiratory symptoms, late-presenting CDH should be considered as a differential diagnosis. Although our study is limited by its retrospective design and small sample size, timely diagnosis and surgery may result in favorable short-term outcomes. Further studies are needed to validate these findings in larger populations and to overcome these limitations.

## Author contributions

**Conceptualization:** Soo-Hong Kim.

**Formal analysis:** Soo-Hong Kim.

**Investigation:** Ayoung Kang, Soo-Hong Kim.

**Project administration:** Ayoung Kang, Soo-Hong Kim.

**Writing – original draft:** Ayoung Kang, Soo-Hong Kim, Hae-Young Kim.

**Writing – review & editing:** Soo-Hong Kim.
